# Hidden burden: Prevalence of anemia in Taif pediatric population

**DOI:** 10.1097/MD.0000000000048672

**Published:** 2026-05-22

**Authors:** Rana G. Zaini, Naglaa M. Kamal, Ajwan M. Alshumrani, Shihanah M. Alshaibani, Shahad N. Al-Shaibani, Bushra F. Alotaibi, Mohammed Alrehaili, Nojood Althubaity, Amal S.H. Alsofyany, Mohammed A.M. Oshi

**Affiliations:** a Department of Clinical Laboratory Sciences, College of Applied Medical Sciences, Taif University, Taif, Saudi Arabia; b Kasr Alainy Faculty of Medicine, Cairo University, Cairo, Egypt; c Clinical Laboratory Sciences Department, College of Applied Medical Sciences, Taif University, Taif, Saudi Arabia; d Biostatistician, Al Hada Armed Forces Hospital, Taif, Saudi Arabia; e Hematology Department, Al Hada Armed Forces Hospital, Taif, Saudi Arabia; f Pediatric Department, Al Hada Armed Forces Hospital, Taif, Saudi Arabia; g Pediatric Department, Gaafar Ibnauf Children’s Emergency Hospital, Khartoum, Sudan.

**Keywords:** anemia, children, Taif

## Abstract

This study aimed to assess the prevalence and characteristics of previously undiagnosed anemia among children in Taif. The objective was to estimate the prevalence of undiagnosed anemia in a broader pediatric population (aged 2–12 years) across 3 hospitals, and to evaluate severity and morphological patterns. A cross-sectional study was conducted across Taif region’s main hospitals serving children; including Taif Children’s Hospital, King Faisal Hospital, and Taif Armed Forces Hospitals. A total of 264 children aged 2 to 12 years, presenting to outpatient clinics or emergency departments for acute, non-hematological conditions, were included. Children with previously diagnosed anemia or chronic diseases were excluded. Blood samples were collected to measure hemoglobin (Hb) levels, and anemia was diagnosed based on age-specific Hb thresholds. Descriptive statistics, chi-square tests, and independent *t* tests were used for data analysis. Out of 264 children, 28 (10.6%) were found to have undiagnosed anemia. The majority (75%) were aged 2 to 5 years, and a higher prevalence was noted among males (67.9%) compared to females (32.1%), though the gender difference was not statistically significant. There was a significant association between younger age and anemia (*P* < .001). Mean Hb levels were significantly lower in anemic children (10.13 ± 0.90 g/dL) than in non-anemic children (13.11 ± 0.93 g/dL; *P* < .001). Most cases were mild (71.4%), followed by moderate anemia (28.6%). Normocytic normochromic anemia was the most common type (53.6%), followed by microcytic hypochromic (39.3%) and macrocytic anemia (7.1%). This study highlights a notable prevalence of undiagnosed anemia among children in Taif, particularly in the preschool age group. The findings underscore the need for routine screening in pediatric outpatient and emergency settings, even in the absence of overt symptoms. Early detection and intervention are essential to prevent long-term developmental complications. Public health efforts should focus on nutritional education, iron supplementation, and comprehensive diagnostic evaluations to address this hidden burden.

## 1. Introduction

Anemia is a multifactorial hematological disorder characterized by a reduction in red blood cell (RBC) mass or hemoglobin (Hb) concentration, impairing the blood’s capacity to deliver oxygen to tissues. In children, the World Health Organization (WHO) defines anemia as a Hb level below 11 g/dL.^[1]^ Insufficient oxygen delivery can result in a spectrum of clinical manifestations, ranging from fatigue and poor concentration to cardiopulmonary symptoms, depending on severity, chronicity, and underlying etiology.^[2]^

Anemia is classified by RBC morphology into microcytic, macrocytic, and normocytic patterns, which reflect common etiologies such as iron deficiency, vitamin B12 or folate deficiency, and anemia of chronic disease, respectively.^[3]^ Additional causes include genetic disorders such as thalassemia and sickle cell disease,^[4]^ blood loss, reduced erythropoiesis due to bone marrow disorders or chronic illness, and increased RBC destruction in hemolytic conditions.^[5,6]^ WHO severity thresholds vary by age, with mild, moderate, and severe anemia defined according to specific Hb cutoffs for children aged 6 months to 12 years.^[1]^

Globally, anemia remains a major public health concern. In 2019, approximately 40% of children aged 6 months to 5 years were anemic, with the highest prevalence observed in the African Region (60.2%).^[7]^ Childhood anemia adversely affects cognitive development, growth, immune function, and academic performance. A study from southern Ethiopia reported a prevalence of 13.2% among children under 5, with more than half presenting with severe anemia.^[8]^ Iron deficiency anemia (IDA) is the most common form worldwide and is strongly influenced by nutritional practices, socioeconomic status, and access to healthcare.

In the Middle East, particularly the Arab Gulf region, IDA remains highly prevalent. Among preschool children, reported rates range from 20% to 67%, with school-aged children exhibiting prevalence between 12.6% and 50%.^[9]^ In Saudi Arabia, despite improvements in living standards, anemia continues to affect a substantial proportion of children. In a study of 2415 children in the southwest region, 26.4% were anemic – predominantly with mild or moderate severity – with microcytic hypochromic anemia being the most frequent morphological type.^[10]^ More recent data from Riyadh showed an overall IDA prevalence of 9.2%, highest among infants and toddlers.^[11]^ Another multicenter study evaluating children at school entry reported a 24% anemia prevalence, with nationality identified as a key determinant.^[12]^ In Taif City, previously undiagnosed anemia among preschoolers was reported at 6%.^[13]^

The present study extends this evidence by examining a broader pediatric age range (2–12 years) across multiple healthcare centers in Taif, thereby providing updated local data on the prevalence and characteristics of undiagnosed anemia in children.

## 2. Methods

### 2.1. Study design and population

A cross-sectional study was conducted across Taif region’s main hospitals serving children; including Taif Children’s Hospital, King Faisal Hospital, and Taif Armed Forces Hospitals. Data was collected from pediatric patients attending outpatient clinics, emergency departments, and inpatient wards during the period from January to March 2025.

All children fulfilling the age inclusion criteria range along with absence of history of anemia or chronic disease during the specified study period were included. Children with previously diagnosed anemia or known chronic illnesses were excluded.

**Figure 1.**
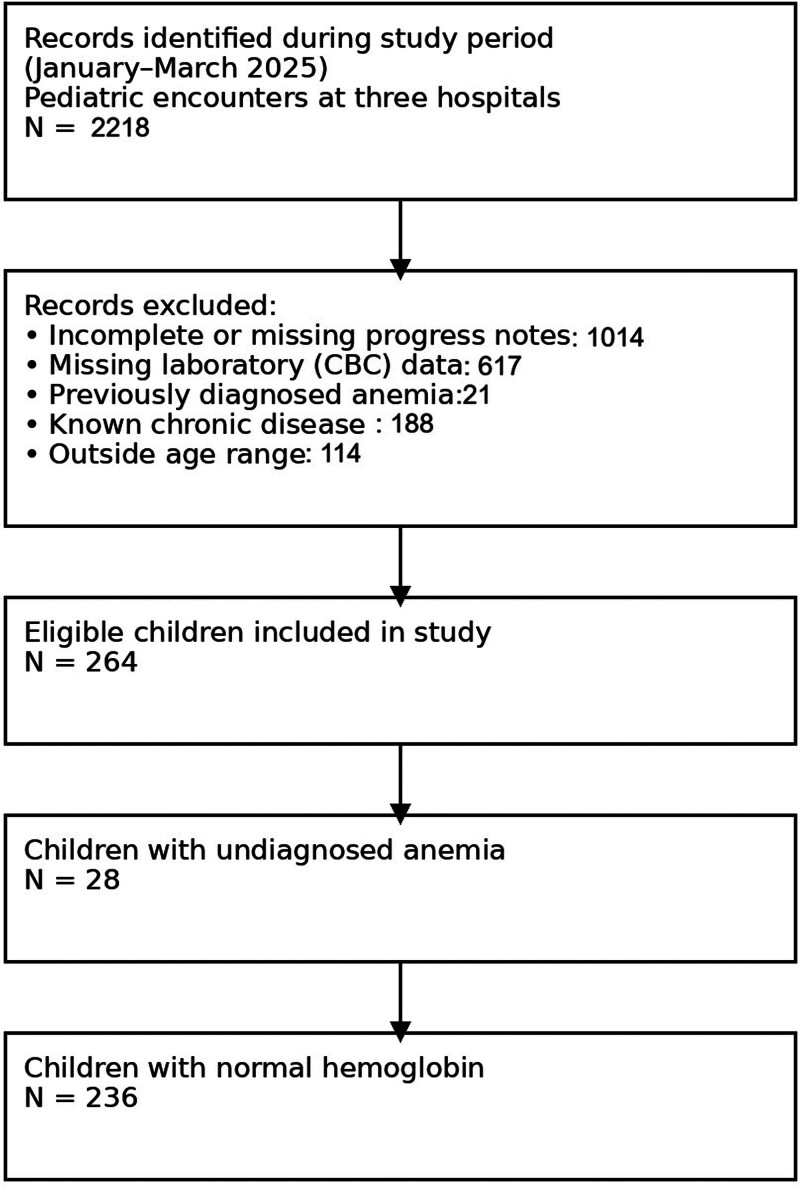
The flow of participant identification, screening, exclusions, and final inclusion.

The study included 264 children, aged 2 to 12 years, who had no prior diagnosis of anemia or history of chronic diseases. The flow chart of participants’ enrollment is shown in Figure 1.

### 2.2. Data collection and laboratory procedures

After obtaining informed written consent from parents or legal guardians, demographic and clinical data including age, gender, nationality, and clinical symptoms – were recorded. Venous blood samples were collected from each child and analyzed at the recruiting Hospital.

Complete blood count (CBC) was performed using the Abbott Alinity hq hematology analyzer on EDTA-anticoagulated samples.

### 2.3. Diagnostic criteria for anemia

Anemia was defined using age-specific thresholds. Mild, moderate, and severe anemia were classified based on WHO criteria, with cutoffs defined by Hb concentration for respective age groups.^[1]^

- For children aged 2 to 5 years and 11 months: Hb < 11.0 g/dL.
- For children aged 6 to 12 years: Hb < 11.2 g/dL.

### 2.4. Statistical analysis

Statistical assumptions for normality and equal variance were checked before applying chi-square and *t* tests. Data were analyzed using descriptive statistics to calculate the prevalence of undiagnosed anemia.

- Chi-square test was used to evaluate associations between categorical variables (e.g., age group, gender) and anemia status.
- Independent *t* tests compared mean Hb levels between children with normal and low Hb values. All analyses were performed using age-specific reference ranges to ensure diagnostic accuracy and standardization.

## 3. Results

This study included a total of 264 children who presented to the emergency departments and outpatient clinics of 3 hospitals in Taif, at Western region of Saudi Arabia: Children’s Hospital, King Faisal Hospital, and Armed Forces Hospital at Al Hada. All participants were free from chronic illnesses that could potentially influence Hb or RBC levels. The children sought medical attention for a variety of acute conditions, such as tonsillitis, constipation, runny nose, allergies, abdominal pain, fever, headache, asthma, diarrhea and vomiting, acute upper respiratory infections, joint pain, eye puffiness, and insect bites.

Among the 264 children enrolled in the study, the majority were Saudi nationals 252 (95.5%). The study population consisted of 154 males (58.3%) and 110 females (41.7%) (Table 1). Undiagnosed anemia was identified in 28 children, representing a prevalence of 10.6% within the study sample. Of these, 19 were males (67.9%) and 9 were females (32.1%), indicating a higher occurrence among boys. However, there was no statistically significant association between gender and the prevalence of undiagnosed anemia among the children in this study (*P* = .283, chi-square test).

**Table 1:** Demographic characteristics of the study population (N = 264).

Variable	Category	Frequency (n)	Percentage (%)
Nationality	Saudi	252	95.5%
	Non-Saudi	12	4.5%
Gender	Male	154	58.3%
	Female	110	41.7%
Age group	2–5 years and 11 months	113	42.8%
	6–12 years	151	57.2%

Regarding the age distribution of anemic patients, 21 children (75%) were between the ages of 2 and 5 years and 11 months, while the remaining 7 children (25%) were between 6 and 12 years old (Table 2). There was a statistically significant association between age groups and undiagnosed anemia (*P* < .001). Children in the younger age group (2–5 years and 11 months) had a significantly higher prevalence of previously undiagnosed anemia compared to older children (6–12 years).

**Table 2:** Descriptive statistics of hemoglobin (Hb) levels among children with normal Hb and undiagnosed anemia.

Group	Mean Hb (g/dL)	Standard deviation (SD)	Sample size (n)
Normal	13.09	0.94	215
Anemic (undiagnosed)	10.13	0.90	28

Values are presented as mean ± standard deviation (SD).

An independent *t* test was used to compare the groups, and a statistically significant difference was observed (*P* < .001).

Most undiagnosed anemia cases (22 out of 28) were detected at Children’s Hospital, accounting for 78.6% of all anemia cases. In contrast, only 3 cases (10.7%) were reported at King Faisal Hospital and 3 cases (10.7%) at Armed Forces Hospital.

This study compared Hb levels among children with undiagnosed anemia and those with normal Hb using an independent *t* test. The mean Hb level among children with normal Hb was 13.11 g/dL with a standard deviation of 0.93 g/dL (n = 215), while the mean Hb level among children with undiagnosed anemia was significantly lower at 10.13 g/dL, with a standard deviation of 0.90 g/dL (n = 28). The independent *t* test revealed a highly statistically significant (*P* < .001) between the 2 groups. This indicates that the difference in mean Hb levels between anemic and non-anemic children is statistically significant (Table 2).

**Figure 2.**
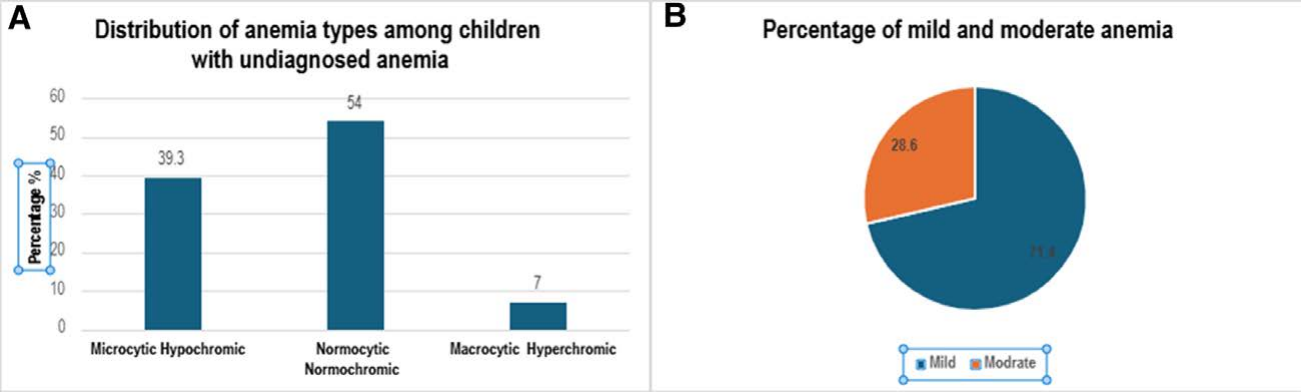
(A) Distribution of anemia types among children with undiagnosed anemia (n = 28). Normocytic normochromic anemia was the most common type (54%), followed by microcytic hypochromic anemia (39.3%), and macrocytic hyperchromic anemia (7%). (B) The percentage distribution of mild and moderate undiagnosed anemia among children.

When analyzing the anemia profiles based on RBC indices, it was found that 15 children (53.6%) had normocytic normochromic anemia, 11 children (39.3%) had microcytic hypochromic anemia, and only 2 children (7.1%) were diagnosed with macrocytic hyperchromic anemia. These findings highlight a noticeable prevalence of undiagnosed anemia among children attending outpatient and emergency services, particularly in the younger age group and predominantly among male children (Fig. 2A). The results of this study also revealed that the majority of children with previously undiagnosed anemia had mild anemia, accounting for 71.4%, while only 28.6% were found to have moderate anemia (Fig. 2B).

## 4. Discussion

This study assessed the prevalence of undiagnosed anemia among children presenting to 3 hospitals in Taif, Saudi Arabia, and found that 10.6% of the participants were anemic, with a significantly higher occurrence among children aged 2 to 5 years. These findings are clinically important, as they highlight a hidden burden of anemia in a population not previously diagnosed or under regular hematological screening.

The results are consistent with the findings of Zaini et al,^[13]^ who conducted a similar study in Taif and reported a comparable prevalence of previously undiagnosed anemia among preschool children. Both studies reinforce the notion that anemia remains underdiagnosed in pediatric populations, particularly in outpatient and emergency settings where hematologic evaluations are not routine. Zaini et al also emphasized the need for proactive screening strategies and improved nutritional interventions, which align with the recommendations drawn from this current study.^[13]^

On a global scale, the observed prevalence aligns with international data. The WHO^[1]^ estimates that anemia affects approximately 42% of children under 5 years worldwide. While the prevalence in this study is lower than in some low-income countries, it still represents a public health concern in Saudi Arabia, especially considering that all cases were previously undiagnosed and could have been missed without targeted screening.

The age-specific distribution of anemia was statistically significant, with children aged 2 to 5 years being disproportionately affected. This trend is supported by global literature, as younger children have higher physiological iron demands due to rapid growth, rendering them more vulnerable to IDA.^[14,15]^ Similarly, Al Zenki et al^[16]^ found high anemia rates among Saudi preschoolers, highlighting a continued nutritional gap despite nationwide supplementation programs.

In terms of gender distribution, more boys than girls were anemic (67.9% vs 32.1%), though the difference was not statistically significant. This is consistent with findings from Balarajan et al,^[17]^ who noted minimal gender differences in pediatric anemia, likely due to the shared physiological demands of early childhood in both sexes.

The majority of anemia cases in this study were classified as normocytic normochromic anemia (53.6%), followed by microcytic hypochromic anemia (39.3%). This pattern contrasts with the typical pediatric distribution where microcytic anemia – largely driven by iron deficiency – tends to predominate. The higher proportion of normocytic anemia in this cohort may reflect several factors. First, many children were evaluated during acute presentations in outpatient and emergency settings, where inflammation and transient physiological stress can suppress erythropoiesis and produce a normocytic profile even when underlying iron deficiency is evolving. Second, early-stage iron deficiency often appears normocytic before microcytosis develops, suggesting that a subset of these cases may represent incipient nutritional deficiency not yet advanced enough to alter red cell indices. Additionally, acute infections – which were common reasons for presentation in this sample – can lead to anemia of inflammation, another normocytic process. A similar limitation was noted by Zaini et al,^[13]^ who emphasized that the absence of iron studies restricts further etiologic classification. Therefore, while microcytic anemia remains clinically expected in children, the predominance of normocytic morphology in this study likely reflects the timing of testing, acute illness patterns, and early or mixed causes of anemia among the included patients.

The results of this study revealed that the majority of children with undiagnosed anemia had mild anemia (71.4%), while 28.6% had moderate anemia. This pattern is consistent with findings from other studies. For example, a study conducted in India by Kaur et al^[18]^ among school-aged children reported that 72% of anemic children had mild anemia, while 26% had moderate anemia. These findings suggest that mild anemia remains the most prevalent form in community-based pediatric populations and may go unnoticed without targeted screening. In contrast, a study conducted in Ethiopia reported an overall anemia prevalence of 53.7% of children participated in the study, out of which, 18.1% had mild while 19.6% of them had moderate anemia.^[19]^ Unlike the current findings, the Ethiopian study observed a slightly higher proportion of moderate anemia compared to mild cases. This difference may be attributed to variations in nutritional status, socioeconomic conditions, healthcare access, or the underlying causes of anemia in each region.

The distribution of previously undiagnosed anemia varied markedly across the participating hospitals. Of the 28 cases identified, Children’s Hospital accounted for the overwhelming majority (22 cases; 78.6%), while King Faisal Hospital and the Armed Forces Hospital each reported 3 cases (10.7%). Although all 3 hospitals served similar pediatric age groups, several factors may explain this imbalance. First, Children’s Hospital likely manages a higher pediatric patient load, particularly for acute and general medical complaints, increasing the opportunity to identify incidental anemia during routine clinical assessments. Higher patient volume naturally raises the likelihood of detecting cases that would otherwise remain unrecognized. Second, screening practices may differ across facilities. Children’s Hospital may have more standardized or proactive use of CBC testing when children present with nonspecific symptoms such as fever, respiratory infections, or gastrointestinal complaints. These presentations comprised much of the study population and often prompt laboratory evaluation, facilitating detection of mild or asymptomatic anemia. Third, the hospital’s role as a primary pediatric care center may attract younger children, the age group shown to have the highest anemia prevalence in this study (75% of anemic cases were aged 2–5 years). In contrast, King Faisal Hospital and the Armed Forces Hospital, which serve broader and more diverse populations, may receive fewer young children or may perform fewer routine screenings in this age group. This uneven distribution does not necessarily indicate true differences in anemia prevalence between hospital catchment areas but more likely reflects variability in patient flow, institutional screening patterns, and case-mix differences. These findings highlight the need for standardized anemia screening protocols across all pediatric care settings in Taif to ensure early detection and consistent management.

Statistical analysis revealed a highly significant difference in mean Hb levels between children with undiagnosed anemia (10.13 ± 0.90 g/dL) and those with normal Hb (13.11 ± 0.93 g/dL) with *P* < .001. This supports the clinical validity of the findings and reinforces the critical need for early identification and intervention. Anemia during early childhood has been linked to long-term negative outcomes, including cognitive delays, impaired immunity, and reduced growth potential.^[20]^

This study has several limitations that warrant consideration. First, the sample was restricted to children presenting to 3 hospitals in Taif, which may limit the external validity of the findings and may not reflect the broader pediatric population across Saudi Arabia. Second, the cross-sectional study design does not allow for assessment of causal pathways or the temporal progression of anemia, including transitions between morphological patterns. Third, the diagnostic workup was limited to CBC parameters; the absence of key biochemical markers – such as serum ferritin, transferrin saturation, soluble transferrin receptor, and inflammatory markers such as C-reactive protein – restricted the ability to determine the specific etiologies of anemia or differentiate between iron deficiency and inflammation-driven anemia. Furthermore, important contextual factors, including dietary intake, socioeconomic status, and parental education level, were not evaluated despite their well-established influence on pediatric anemia risk. Finally, although the overall sample size was adequate, the relatively small number of anemic cases (n = 28) limits the statistical power for subgroup analyses and may reduce the precision of estimates related to anemia subtypes.

## 5. Conclusions

This study highlights the presence of previously undiagnosed anemia among children attending healthcare facilities in Taif, with the highest frequency observed in the preschool age group. While the findings align with prior regional reports, they should be interpreted with caution due to the study’s limited geographic scope, cross-sectional design, and lack of detailed etiologic laboratory markers. The results nonetheless underscore the potential value of incorporating targeted anemia screening, particularly for young children presenting with acute illnesses – into routine outpatient and emergency care pathways. Broader public health efforts aimed at improving nutrition, caregiver awareness, and early recognition may also contribute to reducing the burden of pediatric anemia. Future research should employ comprehensive diagnostic panels and longitudinal designs to better characterize underlying causes and assess long-term outcomes of anemia in Saudi children.

## Acknowledgments

The authors would like to acknowledge the Deanship of Graduate Studies and Scientific Research, Taif University, for funding this work. We also thank the Taif Health Cluster, as well as the Children’s Hospital, King Faisal Hospital, and the Armed Forces Hospital at Al Hada for their assistance in data collection.

## Author contributions

**Conceptualization:** Rana G. Zaini.

**Data curation:** Rana G. Zaini, Naglaa M. Kamal, Ajwan M. Alshumrani, Shihanah M. Alshaibani, Shahad N. Al-Shaibani, Bushra F. Alotaibi, Amal S.H. Alsofyany, Mohammed A.M. Oshi.

**Formal analysis:** Rana G. Zaini, Mohammed Alrehaili.

**Investigation:** Rana G. Zaini, Naglaa M. Kamal, Nojood Althubaity.

**Methodology:** Rana G. Zaini, Mohammed Alrehaili, Nojood Althubaity.

**Project administration:** Rana G. Zaini.

**Software:** Mohammed Alrehaili.

**Supervision:** Rana G. Zaini.

**Validation:** Rana G. Zaini.

**Visualization:** Rana G. Zaini.

**Writing – original draft:** Rana G. Zaini, Ajwan M. Alshumrani, Shihanah M. Alshaibani, Shahad N. Al-Shaibani.

**Writing – review & editing:** Rana G. Zaini, Naglaa M. Kamal, Mohammed A.M. Oshi.
